# Patient perceptions of success in obesity treatment: An IMI2 SOPHIA study

**DOI:** 10.1002/osp4.70001

**Published:** 2024-08-17

**Authors:** E. Farrell, J. Nadglowski, E. Hollmann, C. W. le Roux, D. McGillicuddy

**Affiliations:** ^1^ University College Dublin Dublin Ireland; ^2^ Obesity Action Coalition Tampa Florida USA; ^3^ School of Medicine University College Dublin Dublin Ireland; ^4^ School of Education University College Dublin Dublin Ireland

**Keywords:** obesity treatment, patient experience, qualitative, success

## Abstract

**Background:**

It is anticipated that by 2030, 20% of the world's population will live with obesity. Success in the management of obesity is predominately determined in terms of BMI or percentage weight loss, yet the limitations of these have been widely recognized. This study aimed to understand patient definitions of success in obesity treatment.

**Methods:**

A series of in‐depth focus groups, carried out with n = 30 adults living with obesity, offered a qualitative insight into patient definitions of success.

**Results:**

A thematic analysis of data yielded four thematic findings: Success as freedom from stigma, bias and the mental burden of obesity; success as being able to participate fully in the world; success as measured by NSVs [non‐scale victories]; and success is not a number on a scale.

**Conclusions:**

What this study highlights is (1) how current measures of success do not accurately encompass the priorities of people living with obesity, (2) the importance of addressing the psychological and emotional aspects of living with obesity in any definition of success , and (3) the importance of meaningful co‐creation of goals and indicators of success between clinician and patient for the effective management of the disease of obesity.

## 1 INTRODUCTION

There is currently no standard definition of the successful management of obesity.^1^ Clinical practice guidelines for the management of obesity rely instead on indicators such as weight loss or a reduction in BMI.^1^ Indeed, a recent synthesis of measures of success in clinical practice guidelines published in this journal found that “the most common measures of success were defined as weight loss (e.g.,% body weight)or health outcomes.”^1^ For example, the recently updated NICE clinical guideline for obesity^2^ measures success in terms of BMI, waist‐to‐height ratio and health outcome. Such measures of success have been subject to criticism with the American Medical Association recently describing BMI as “an imperfect clinical measure.”^3^ However, an agreed definition of success is required to evaluate the efficacy of a treatment or intervention and, at an individual level, to establish a shared opinion of successful disease management. Without a shared understanding of what “success” looks like, there is a risk that patients will lose trust in their clinicians and become frustrated with their program or progress. In the absence of patient‐generated definitions of success, this study sought to understand patient understandings of success in obesity treatment and management.

## 2 MATERIALS AND METHODS

### 2.1 Study design

A qualitative interpretative research design was performed including a focus group methodology to generate data on the lived experience of obesity treatment. In contrast to individual or group interviews, focus groups encourage interaction between participants: asking questions, sharing experiences, and expanding similarities and differences in points of view. As such, focus groups are particularly effective in exploring people's knowledge and experiences and offer an insight into “not only what people think but how they think and why they think that way.”^4^ Ethical approval for this study was granted by UCD's Human Research Ethics Committee (HS‐20‐12‐McGillicuddy).

### 2.2 Recruitment and participants

Participants were recruited based on their experience of obesity treatment. We sought to include participants at the early stages of their treatment journey as well as those more experienced in accessing support and care for obesity. To do this, we recruited participants at the early stages of their engagement with one Irish hospital‐based weight management clinic (n = 7) as well as participants from leading European and US obesity advocacy organizations Obesity Action Coalition (OAC), European Coalition for People Living with Obesity (ECPO), and Irish Coalition for People living with Obesity (ICPO) (n = 23). Participants were not known to the interviewers in advance of the study commencement. The sample included 11 men and 19 women, of which 24 were European and six were based in the US or Canada. Table 1 provides further details on the sample as well as an overview of the treatment access by participants. Each participant has been assigned a pseudonym in order to protect their anonymity.

**TABLE 1:** Sample characteristics and overview of treatments undertaken by participants.

	Sex	Age range	Self‐directed behavior modification	HCP‐led behavior modification	Meal replacements	Community‐based/Non‐medical programmes	Commercial weight loss programmes	Supplements	Medications (prescribed)	Bariatric surgery
Amanda	F	40–49	X	X	X	X	X		X	X
Anna	F	40–49					X			X
Bob	M	60–69	X	X	X					X
Brianna	F	50–59	X	X	X	X	X		X	X
Caroline	F	50–59								X
Camila	F	70–79	X	X	X	X	X			
Damian	M	50–59						X	X	
Daniella	F	50–59	X	X	X	X	X	X		
Eddie	M	40–49	N/A	N/A	N/A	N/A	N/A	N/A	N/A	N/A
George	M	60–69	X	X	X	X			X	
Graham	M	60–69	X	X	X	X	X			X
Harper	F	40–49							X	
Isla	F	50–59	N/A	N/A	N/A	N/A	N/A	N/A	N/A	N/A
Kimberly	F	40–49	X	X	X	X	X	X		X
Layla	F	30–39	N/A	N/A	N/A	N/A	N/A	N/A	N/A	N/A
Lydia	F	40–49	X	X		X	X		X	
Melanie	F	30–39	X	X		X	X	X	X	X
Max	M	50–59	X	X		X	X	X	X	X
Maeve	F	60–69				X			X	X
Matteo	M	30–39	N/A	N/A	N/A	N/A	N/A	N/A	N/A	N/A
Olivia	F	30–39	X	X		X	X			X
Ronan	M	40–49	X	X			X		X	X
Samantha	F	20–29							X	X
Stella	F	50–59		X					X	
Selina	F	30–39							X	X
Susanna	F	50–59	X	X	X	X	X	X	X	
Sonia	F	40–49	X	X	X	X	X	X	X	X
Taylor	M	60–69		X						
Vihaan	M	30–39	X		X	X		X		
Walter	M	70–79	X	X	X					

### 2.3 Data Generation

Online focus groups were carried out by two experienced researchers (EF and EH) between April and July 2022. Each focus group ranged in size from two to four persons and was scheduled at a time convenient for participants and their respective time zones. In advance of each focus group, participants were sent an information sheet and consent form, which they had time to read and discuss with researchers if they had any questions or concerns. Participants were also invited to complete a brief online questionnaire which asked them to indicate the types of treatment they have tried to date. These data are presented in Table 1. The purpose of the focus group was to understand patients' experience of obesity treatment and, in this instance, specifically their understanding and definition of success in obesity treatment. Each group was offered the same question—what does success mean to you? The interviews ranged in duration from 40—90 min. Each participant was offered the opportunity to share their own personal conceptualization of success, with each account sparking resonance and remembrance in the other participants present and thus yielding a rich and layered conversation.

### 2.4 Reflexivity statement

This research was conducted as part of a larger study (https://www.imisophia.eu) led by ClR with the objective of optimizing the future of obesity treatment. Within this objective, the “patient preferences” work‐package team (JN, DMcG, EF, EH) were free to be guided by the patients we worked with who helped us recognize key aspects of their experience that remain under‐represented in the scholarly literature. Early open‐ended phenomenological interviewing foregrounded the finding that “success”, for people with obesity, was not best reflected in the BMI‐centric definitions used by their HCPs. The team decided to examine this further by inserting a specific question on success into the focus group interview schedule, yielding the data presented in this paper. In recognition that the early phenomenological interviews were carried out with Irish adults living with obesity, we decided to broaden the sample to include the voices and perspectives of people living with obesity in North America and Europe. This paper was led by EF, whose interest in hermeneutic phenomenological health research evokes an emphasis on “the announcement and making known in language the being of a being (*Dasein*) in its being”.^5^ JN provided an informed sounding board whose expertise in leading the world's largest patient advocacy organization as well as personal and family experience with obesity helped locate the findings within the broader debate within the field of obesity. The team's interdisciplinary expertise, which includes medical (ClR), psychological (EF), advocacy (JN), qualitative research (DMcG) and nursing (EH) expertise, provided a rich and diverse disciplinary context in which the knowledge presented here was generated.

### 2.5 Data analysis

All online focus group discussions were recorded and transcribed verbatim and initial analysis was completed by one researcher (EF) using Braun and Clarke's^6^ six‐step framework for thematic analysis. This framework involved (a) becoming familiar with the data by reading and rereading the data in their entirety; (b) generating initial codes by jotting down descriptive labels and key words; (c) taking these initial codes and sorting them into potential themes; (d) reviewing and refining these prospective themes in terms of how accurately they reflected the meanings in the data; (e) further refining and defining these themes and what they revealed about participants perceptions of the risks associated with obesity treatment; and (f) formulating the findings as a “whole” and presenting them as described below. The wider research team (JN, EH, DMcG) were presented with findings at step (d) and step (f) and the ensuing discussion facilitated a degree of consensus and honed the thematic findings presented below.

## 3 RESULTS

Participant conceptualizations of success are organized below under four thematic headings: Success as freedom from stigma, bias and the mental burden of obesity; success as being able to participate fully in the world; success as measured by NSVs [non‐scale victories]; and success is not a number on a scale. These themes are semantic themes in that they were identified through the explicit meanings of the data.

### 3.1 Success as freedom from stigma, bias and the mental burden of obesity

Freedom was a recurring theme across the focus groups carried out—particularly freedom from the stigma, bias and discrimination associated with obesity. Lydia spoke about the mental “burden” of living with obesity:

When I think of success, the word that comes to mind is freedom. And its freedom to go places and to do things without burden. There's the obvious burden of the weight that you carry, but there's also the mental weight that you carry too. Wondering if there's space for me, or wondering who might say something, or what might happen, or if I will fit in. Wondering if I'm going to have to endure a conversation again and wondering if I'm going to be discriminated against. Just freedom to just be—Lydia.

She also described a strong desire to be free of the burden of bias, discrimination and “feeling out of place:”

Because, no matter what number might be on the scale, if you fall into that obese category, you have dealt with weight bias, you have dealt with discrimination at the doctor's office, you have dealt with feeling out of place in school, in your office, in society in your own head, in your family. And success for me looks like freeing myself and others from that—Lydia.

Melanie too described success in terms of freedom from the burden of “the thoughts that go with it [obesity]:”

It's like a freedom from it. It's like a freedom from the thoughts that go with it. The constant thinking, the constant thinking about what food, what I weigh, what I wear, and what I look like. It's all connected—Melanie.

### 3.2 Success as being able to participate fully in the world

Similar to the theme of success as freedom from the stigma and mental “burden” of living with obesity, many participants conceptualized success in terms of being able to fully participate in the world.

I think the marker for success has definitely shifted, especially in the past two to 3 years with there being a wider range of acceptance of body types. So, you don't necessarily have to be a size six or 10, or any specific size ‐ success is being in a body that allows you to exist happily, that allows you to exist freely, that allows you to exist in the moment. And I think for many people, it's not losing a certain amount of pounds, but am I able to participate fully in the world—Kimberly.

To be able to move better, to be able to participate better in things, be able to… just your life would be made easier—Camilla.

Kimberley: Success is being in a body that allows you to exist happily, that allows you to exist freely, that allows you to exist in the moment. And I think for many people, it's not losing a certain amount of pounds, but am I able to participate fully in the world.

Researcher: And what does that look like?

Kimberley: That looks like being able to go to a restaurant and sit in a booth, without worrying about am I going to fit. It's about putting on a bright colored outfit and not worried about people calling you the Kool‐Aid Man. It looks like being able to just exist authentically in the world.

Being able to participate fully was associated, for many, with simply being able to do the things they wanted to do.

I would say that success is not about the number on the scale, success is about all of the things that you can do that you couldn't do before.—Max.

Whatever size I end up being, it's what I'm going to be able to do, and it's what I'm going to be able to do with my life, I think that's more of a metric of success than… a lot of times, we start that weight loss journey, it's like, oh, well then you need to lose 10%, or 20%, or this many pounds. But I think for me, success would be what I can do.—Vihann

I think the biggest success is being able to put in a hard days' work and get up the next morning and being able to walk. So, whereas before, if I put in a hard days' work with a lot of walking and lifting and stuff and climbing ladders and that, the next morning I wouldn't be able to walk. For a couple of hours, I'd be in pain. But now I get up, and within 15 to 20 min, it's gone. So, that's a huge success—Graham.

### 3.3 Success as measured by NSV's [non‐scale victories]

The more everyday indicators of success, which participants described as NSVs, or non‐scale victories, were considered as important to participants as the, more celebrated, scale victories.

[It's] not just the number on the scale but being able to fit into that roller coaster or tie your shoelaces without having to hold your breath. In our communities, we call them NSVs, which is short for non‐scale victories—Layla.

It's always uplifting to hear these [NSVs}. And they're really… I think they're really poignant, because there are a lot of things that folks would take for granted, you know, like the tying my shoelaces one is a popular one. But even something as simple as a sitting and crossing my legs ‐ you know, most people would [say] why are you so excited about crossing your legs? But for a lot of us, we understand what it takes to get there and not being able to do that. One of the most beautiful ones that I've ever heard and I love hearing this one—a mother had shared with me that it took 14 years for her daughter to finally be able to give her a hug and her fingers touch in the back. And I think about that one quite often. But these moments of success, you know, celebrate our humanity, and more than just that number on the scale—Vihann.

When the subject of NSVs came up in a focus group, it inevitably stirred a dynamic conversation about each person's own NSV, as demonstrated below:

Olivia: Everyone has probably different ones [NSVs]. I know one of mines was fit into Penney's [high street] clothes, because I hadn't fit into Penney's clothes in years. And this one [points to picture] ‐ I have it framed in the kitchen, this is my success—the seatbelt went around me going to Portugal and I have it framed.

Maeve: That's the success story, the seatbelt on the plane

Eddie: I can't wait for this seatbelt to go around me. It's like, in the car, I used to always put the safety belt at the back of me ‐ but now the safety belt comes over me, so my next trip is the plane.

Maeve: That is another thing, sitting on the rides with the kids

Eddie: Or on the trains. When I used to go on the trains, I'd always sit at the back seats, where there were no [fixed] tables, just the pull‐down tables. But I was on a train there 2 weeks ago, and I was able to fit into the seats properly. I used to often have to stand up and the train rather than sitting.

### 3.4 Success is not a number on a scale.

One theme that came up time and again was participants insistence that success is greater than a number on a scale:

I don't have a set idea of success in my head, but I know it's not a particular weight—Harper.

I would say that success is not about the number on the scale, success is about all of the things that you can do that you couldn't do before. Success is about health. Success is about living longer than you thought you were going to live—Max.

Success for me was losing a huge amount of weight [but then] I came to realize that I didn't need to be rake thin to be successful. That wasn't what success was—Ronan.

I think the initial success is deemed to have got to 25% of your heaviest weight, losing that much or whatever. But [really] it's about wherever your weight settles, learning that's where your body needs to be and learning to be happy in that and being healthy at that weight and that is there any genuine success?—Samantha.

It is important to acknowledge, however, that weight was the mark of success for one participant:

Well, my success is getting down to the size I am. I never thought I could get down to this size before and it's just great—Damian.

## 4 DISCUSSION

This study sought to understand patient conceptualizations of success in obesity treatment. Success in the management of obesity “is an inherently subjective and complex concept.”^1^ Indeed, success or a definitive endpoint in the management of any complex chronic relapsing disease is difficult to reify or define.^7^, ^8^, ^9^ In the face of such subjectivity and complexity, standard measures such as BMI or weight loss are employed as inexpensive and easily quantifiable endpoints by which interventions can be assessed and associated with other clinical outcomes.^3^

However, such measures of success have been subject to criticism, with the American Medical Association recently describing BMI as “an imperfect clinical measure.”^3^ While significantly correlated with the amount of fat in the general population, BMI loses predictability and applicability when applied at an individual level.^10^ This study highlights how people living with obesity also consider weight loss or BMI as imperfect measures of success, with just one of this study's 30 participants defined success in terms of weight loss. Other participants, all of whom have lived experience of obesity and obesity treatment, consider success as being much more than “the number on a scale” (Max). Yet, a recent qualitative synthesis of 16 leading clinical practice guidelines for obesity revealed that weight loss (e.g., % body weight) remains the most common clinical measure of success.^1^ For example, the recently updated NICE clinical guideline for obesity^2^ measures success in terms of BMI, waist‐to‐height ratio and health outcome. The Singapore G14 guideline cautions against “normalization of BMI [as] not a realistic goal. Instead, an initial goal of 5%–10% weight loss over 6 months will help reduce comorbidities significantly.”^11^

Whether determined by BMI or percentage weight loss, defining success in terms of clinical measures reflects a second difficulty with common indicators of success in obesity management—the definition of success as controlled by the professional and not the patient. In their synthesis of measures of success as defined by clinical practice guidelines for obesity, Juul‐Hindsgaul et al, highlighted how “the underlying assumption [is] that the professional is in control.”^1^ They found that, at best, “devolving control to patients was only in terms of certain aspects of healthcare, and always in alignment with clinicians' views and support.”^1^ Patient definitions of success, particularly those developed independently of clinicians views and support, remain unchartered in obesity research and practice. As such, this study represents the first known patient‐led conceptualization of success in obesity management.

Participants in this study offer a rich insight into their conceptualizations of success. Chief amongst these was an understanding of success as freedom from the stigma, prejudice, bias and mental “burden” (Lydia) associated with the disease. This finding further highlights the deleterious effects of weight bias, stigma and discrimination on the health and well‐being of people living with obesity.^12^, ^13^, ^14^ It suggests that any definition of success ought to consider psychological and emotional indicators of success as much as physical or biological indicators. Psychological therapies, such as CBT, have been shown to be an effective component of the management of obesity.^15^ This study's themes of success as freedom from stigma, and success as being able to participate freely in the world serve to further emphasize the importance of the psychological and emotional dimension of any obesity management plan and/or definition of success. Finally, participants in this study spoke at length about NSVs and non‐scale victories. The sample data presented above offer an insight into the range and import of success when defined in terms of being able to tie one's own shoelaces, travel on an aeroplane, or hug one's child. While these may seem trivial, even insignificant, in comparison to standard measures of obesity success, what this study reveals is the pride, significance and accomplishment associated with NSV for people living with obesity. Overall, this study offers a useful insight into patient conceptualizations of success in obesity treatment and management. It further serves to reinforce the subjectivity and complexity of defining success in obesity management and calls for clinicians and researchers alike to acknowledge, if not embrace, the complexity and subjectivity of success for each individual patient with whom they work. As participants in this study remind us, success is more than a number on a scale and the effective management of obesity begins with the development of a shared understanding of what success looks like for that particular patient at that particular time. In this way, clinicians and patients can work in a collaborative and targeted manner toward the effective realization of that person's individual outcome of success.

## CONFLICT OF INTEREST STATEMENT

Carel le Roux has served on advisory boards for Novo Nordisk, GI Dynamics, Keyron, Sanofi, Boehringer Ingelheim, Herbalife, Johnson and Johnson, whilst receiving grant funding from Science Foundation Ireland, The Health Research Board and the Irish Research Council. Joe Nadglowski is an employee of the OAC. The other authors have no conflicts of interest to report.
